# A Novel Reference for Bt-Resistance Mechanism in *Plutella xylostella* Based on Analysis of the Midgut Transcriptomes

**DOI:** 10.3390/insects12121091

**Published:** 2021-12-07

**Authors:** Lei Xiong, Zhaoxia Liu, Lingling Shen, Chao Xie, Min Ye, Zeyun Li, Zhen Zhang, Jingge Li, Yi Dong, Minsheng You, Shijun You

**Affiliations:** 1 State Key Laboratory of Ecological Pest Control for Fujian and Taiwan Crops, Institute of Applied Ecology, Fujian Agriculture and Forestry University, Fuzhou 350002, China; 18770912289@163.com (L.X.); zhuben1990@126.com (Z.L.); slingling2@126.com (L.S.); 15927295552@163.com (C.X.); ymbjwbjt@163.com (M.Y.); lzy2645693422@163.com (Z.L.); zhangzhen19980718@163.com (Z.Z.); jg_l_email@163.com (J.L.); Dongyi_0106@163.com (Y.D.); msyou@fafu.edu.cn (M.Y.); 2 Ministerial and Provincial Joint Innovation Centre for Safety Production of Cross-Strait Crops, Fujian Agriculture and Forestry University, Fuzhou 350002, China; 3 Joint International Research Laboratory of Ecological Pest Control, Ministry of Education, Fuzhou 350002, China

**Keywords:** *Plutella xylostella*, transcriptome, Cry1Ac, differentially expressed gene, resistance mechanism, *Bacillus thuringiensis*

## Abstract

**Simple Summary:**

*Plutella xylostella* is a very serious pest to cruciferous vegetables. At present, the control methods used are mainly traditional insecticides and the cultivation of Bt crops. However, with the long-term and large-scale use of insecticides, the diamondback moth has developed strong resistance to many kinds of insecticides and Bt crops. The Cry1S1000 strain of *P. xylostella* used here is a strain with more than 8000 times resistance to Bt Cry1Ac protoxin. In this paper, we used transcriptome sequencing to determine the midgut transcriptome of the G88-susceptible strain, Cry1S1000-resistant strain and its corresponding toxin-induced strains to find more genes related to Bt resistance. Our results will provide a reference for optimizing the control strategy of diamondback moth resistance and improving the control efficiency of biopesticides and Bt crops.

**Abstract:**

The diamondback moth, *Plutella xylostella*, is a lepidopteran insect that mainly harms cruciferous vegetables, with strong resistance to a variety of agrochemicals, including *Bacillus thuringiensis* (Bt) toxins. This study intended to screen genes associated with Bt resistance in *P. xylostella* by comparing the midgut transcriptome of Cry1Ac-susceptible and -resistant strains together with two toxin-treated strains 24 h before sampling. A total of 12 samples were analyzed by BGISEQ-500, and each sample obtained an average of 6.35 Gb data. Additionally, 3284 differentially expressed genes (DEGs) were identified in susceptible and resistant strains. Among them, five DEGs for cadherin, 14 for aminopeptidase, zero for alkaline phosphatase, 14 for ATP binding cassette transport, and five heat shock proteins were potentially involved in resistance to Cry1Ac in *P. xylostella*. Furthermore, DEGs associated with “binding”, “catalytic activity”, “cellular process”, “metabolic process”, and “cellular anatomical entity” were more likely to be responsible for resistance to Bt toxin. Thus, together with other omics data, our results will offer prospective genes for the development of Bt resistance, thereby providing a brand new reference for revealing the resistance mechanism to Bt of *P. xylostella*.

## 1. Introduction

The diamondback moth, *Plutella xylostella*, is an important pest of cruciferous crops worldwide [1]. The total economic cost of its damage and management worldwide is more than US $4–5 billion per year [1], primarily due to strong resistance to multifarious synthetic- and bio-insecticides. As the first species that developed resistance to Bt toxins in outdoor populations [2], *P. xylostella* has evolved resistance to all major insecticides and has become increasingly difficult to control. Hence, there is an urgent need to identify the complete mechanisms underlying Bt resistance.

Many different hypotheses have been proposed for the mechanism of action of Bt toxins: the pore formation model [3,4,5], the signal transduction model [6], the direct action model [7], and the dual model [8,9]. The pore formation model believes that the activated toxins in the form of monomers bind to the receptors on the brush border membrane vesicle (BBMV) of the insect midgut, leading to the oligomerization of the monomers. The oligomers then attach to other midgut receptors and eventually insert irreversibly into the membrane, forming perforations, which cause the larvae to die [3,4,5]. In contrast, the second model suggests that Cry protein first binds to cadherin, which activates the signal death pathway in the cell. The Mg^2+^-dependent signaling pathway indicates that, after the binding of Cry toxin to the BT-R site, it stimulates both G protein and adenylate cyclase (AC), promoting intracellular cyclic adenylate (cAMP) levels. In turn, the activation of protein kinase A (PKA) destroys the stability of the cytoskeleton and ion channels, leading to death of cell [6]. In addition, Vachon [7] revealed that activated Cry toxins could be directly inserted into the intestinal membrane after binding to APN, ALP, or cadherin, and oligomerization occurs on the membrane to form pre-pore oligomers, leading to the death of insects. However, contrary to the classical model of action, relevant studies have found that Cry1A protoxin can directly bind to cadherin, resulting in oligomerization in the presence of intestinal protease. Unlike the activated toxin, the pre-pores formed by the protoxin oligomers are more heat-resistant, SDS-resistant, easier to insert into artificial vesicles, and have a stronger ability to create pores. Therefore, scholars call it a double-acting model of Cry toxin; that is, Cry1A protoxin can directly bind to cadherin and oligomerize without activating proteases, forming pre-pore oligomers. Subsequently, it binds to APN or ALP and eventually inserts into the intestinal membrane, leading to perforation of the intestinal membrane that leads to the insect’s death [8,9]. In recent years, Guo et al. [10] conducted more studies on the MAPK signaling pathway. They found that the MAPK signaling pathway can regulate ALP and ABCC gene expression levels in the middle intestine, thus leading to resistance to Bt Cry1Ac toxin on *P. xylostella*. In subsequent studies [11], starting with the potential role of the APN gene, they also confirmed that differential expression of APN and other midgut genes mediated by MAPK is indeed closely associated with resistance. Equally important, they found that the MAPK cascade is stimulated and regulated by partial hormones, suggesting a new mechanism of hormone involvement in Bt resistance. Further research on the three potential activation pathways of the complex four-layer MAPK signaling module provides a good direction for the control of the agricultural pest *P. xylostella* [12].

Currently, more than four Cry toxin receptors have been reported, namely aminopeptidase N (APN) [13,14,15,16,17], cadherin-like (CAD) [18,19,20,21,22], alkaline phosphatase (ALP) [10,23,24,25,26], ATP-binding cassette (ABC) transporters [27,28,29,30,31,32,33,34,35,36], and others. Glycolipid [37], actin [38], and heat shock protein [39] have also been reported as the receptors of Bt, but there are few related reports. The main cause of insect resistance may be the change in receptor protein structure [34] or the difference in the expression level of related genes [40] so that toxin binding is reduced or no longer able to bind with the toxin.

RNA-Seq is a very powerful tool commonly used in the study of the molecular mechanism in pest resistance recently [41,42]. In particular, next-generation sequencing technology has been used by many researchers to identify genes associated with insect resistance, including *P. xylostella* [43], *Chilo suppressalis* [44], *Helicoverpa armigera* [45], *Ostrinia furnacalis* [46], *Busseola fusca* [47], and *Helicoverpa zea* [48]. In addition, RNA-Seq technology makes the analysis of unknown genes and splice variants easier and more feasible. In addition, as the cost of sequencing has fallen, RNA-Seq technology has become more common, including in species whose genomes have not been sequenced [42].

Many studies have been conducted to examine transcriptome of *P. xylostella*, such as tissue-specific transcriptome analysis of a Bt-resistant strain [49]. Comparative studies were also reported on transcriptomic differences between Bt-susceptible and -resistant strains, although the focus is on different Bt toxins [43,45,46,47,50,51]. Moreover, transcriptome analyses of different insecticides and resistant strains have been reported [52,53,54,55]. All this research provides valuable candidate gene resources for studying Bt resistance in insects. For the G88 and Cry1S10000 strains used in this research, similar studies have been carried out in the past. Lei et al. [43] used transcriptome analysis to compare the difference in midgut transcriptome between two resistant strains and one susceptible strain. One resistant strain used in this study had the same source as the resistant strain used in that study. However, the difference was that the resistant strain used in their study had been screened for toxin Cry1Ac in each generation, while the resistant strain used in this study remained highly resistant to Bt Cry1Ac toxin after only one screening with Cry1Ac toxin. Later studies [33,56] on the same genes (*PxABCC2* and *PxABCC3*) also produced different results, so we believe that the resistance strains used in these two studies should have great differences and that our study results provide rich candidate gene resources for studying Bt resistance.

In this study, the transcriptomes of G88-susceptible and Cry1S1000-resistant strains were compared by RNA-Seq. The protoxin-treated strains were also compared to eliminate the expression differences of gene transcription levels caused by toxin induction. In addition, DEGs were further identified and validated by quantitative real-time PCR (qRT-PCR) by comparing both strains [33]. The results of this study provide candidate genes for the evolution of resistance to Bt and a reference for revealing the resistance mechanism to Bt in *P. xylostella*.

## 2. Materials and Methods

### 2.1. Insect Rearing and Sample Collection

The two *P. xylostella* strains (G88 and Cry1Ac-R) were provided by Dr. Anthony M. Shelton in 2016 [57]. Among them, the Cry1Ac-R strain was screened by 1000 μg/mL Cry1Ac protoxin and was never exposed to insecticide again. Here, we used Cry1S1000 to name the Bt resistant strain, as previously reported [33]. Larvae were fed an artificial diet at 26 ± 1 °C, 60 ± 5% RH (relative humidity), and 16:8 h (light:dark) photoperiod. During the adult mating stage, 10% honey water was used for supplemental nutrition. In this study, the resistance ratio of Cry1S1000 to Cry1Ac was >8000-fold compared to the G88 strain. The fourth-instar larvae of both strains were dissected to obtain the midgut tissues. In addition, 24 h before sampling, both strains were fed an artificial diet containing a lower concentration (0.006 µg/mL, LC_30_ of G88 strain) of Cry1Ac protoxin to eliminate toxin-induced changes in transcription levels and ensure that most larvae were alive before sampling. These strains were also dissected to obtain midgut tissues.

### 2.2. Cry1Ac Protoxin Purification

Cry1Ac protoxin was produced by Btk strain HD73. The Bt culture was cultured in 1/2 LB (1 L:2.5 g yeast extract; 5 g tryptone; 5 g NaCl) at 230 rpm for 84 h [58,59]. Bacterial cells were suspended in 1 M NaCl, then centrifuged and washed twice with distilled water to prepare protoxin crystals. Then, the protoxin crystals were solubilized in the lysate (50 mM Na_2_CO_3_; 50 mM EDTA; PH 9.5) with 5% *β*-mercaptoethanol. After centrifugation, the dissolved Cry1Ac protoxin was collected from the supernatant, and then 1/7 volume of 4 M sodium acetate (PH 4.5) was added to the precipitate. After two washes with distilled water, the precipitated protoxin was centrifuged and resuspended in 50 mM Na_2_CO_3_ (PH 9.5). The Bradford method was used to determine the protoxin concentration by using BSA as a standard, and the quality of soluble protoxin was analyzed by 12% SDS-PAGE [60].

### 2.3. RNA Extraction, Library Construction, and Illumina Sequencing

Total RNA was extracted from the susceptible (G88) and resistant (Cry1S1000) strains, as well as from two protoxin-treated groups, before sampling using Trizol reagent (Invitrogen, Carlsbad, CA, USA), according to the manual’s instructions. RNA samples were only used if they had a 260/280 ratio from 1.9 to 2.1 and an RNA integrity number (RIN) higher than 8. The quantity and quality of the total RNA were assessed by a Nano Drop2000 (Thermo Fisher Scientific, Waltham, MA, USA) and Agilent 2100 bioanalyzer (Agilent, Palo Alto, CA, USA). Then, oligo (dT)-attached magnetic beads were used to purify Poly (A) mRNA. The purified mRNAs were segmented with fragment buffer at an appropriate temperature. Then, the first-strand cDNA was generated by reverse transcription using random hexamer-primes, and the second-strand cDNA was synthesized. Afterward, the short fragments were then connected to the sequencing adapters. After agarose gel electrophoresis, appropriate fragments were selected as templates for PCR amplification. Finally, Illumina HiSeq^TM^ 2000 (BGI, Shenzhen, China) was used to sequence the library.

### 2.4. Bioinformatics Analysis of the Transcriptome

The raw data obtained by sequencing were called the raw reads. To obtain high-quality, clean reads for subsequent de novo assembly (Software: SOAPnuke, Version: v1.5.2, Parameter:-l 15-q 0.2-n 0.05), we filtered out the raw reads with low quality, contaminated linkers, and unknown bases [61]. Clean reads were then compared to the reference genome (*P. xylostella* DBM V2, http://iae.fafu.edu.cn/DBM/) (accessed date: 15 October 2021) (Software: HISAT2, Version: v2.0.4, Parameter: --phred64 --sensitive --no-discordant --no-mixed-I 1-X 1000), followed by new transcription prediction (Software 1:StringTie, Version:v1.0.4, Parameter:-f 0.3-j 3-c 5-g 100-s 10,000-p 8; Software 2: Cufflinks, Version: v2.2.1, Parameter: -p 12; Software 3: CPC, Version: v0.9-r2, Parameter: default), SNP and Indel, and differential splicing gene detection (Software 1: Asprofile, Version: b-1.0.4, Parameter: default; Software 2: rMATS, Version: v3.0.9, Parameter: -analysis U-t paired-a 8; software 3: Circos, Version: v0.69, Parameter: default). Next, transcripts with protein-coding potential were added to the reference gene sequence (Software: Bowtie2, Version: v2.2.5, Parameter: -q --phred64 --sensitive --dpad 0 --gbar 99,999,999 --mp 1,1 --np 1 --score-min L, 0,-0.1-p 16-k 200) to form a complete reference sequence. Gene expression level was then calculated, and the DEGs were detected according to the requirements of multiple samples. Finally, the DEGs were further analyzed by in-depth clustering analysis and functional enrichment analysis [62].

### 2.5. Gene Function Annotation and Characterization

The BLASTx search was carried out in protein databases, including KOG (eukaryotic Orthologous Group database), Nr (non-redundant) protein database, SwissPort, and KEGG (Kyoto Encyclopedia of Genes and Genomes protein database), to determine the functional annotation. Furthermore, a BLASTn search was performed using the Nt database. The InterPro annotation and GO (Gene Ontology) of unigenes were acquired using the Blast2GO and InterProScan5 program with Nr annotation, respectively [63]. Then, GO classification was performed to elucidate the distribution of DEG functions, including biological process, cellular component, and molecular function [64]. According to the annotation results of GO, KEGG, and official classification, functional classification of the differential genes was performed, and Phyper R in R (v.3.6.1) software was used for enrichment analysis. The *p*-value calculation method is as follows:

(1) $$P\;=1-\overset{m-1}{\underset{i=0}{\sum }}\frac{\left(\begin{array}{c} M\\ i \end{array}\right)\left(\begin{array}{c} N-M\\ n-i \end{array}\right)}{\left(\begin{array}{c} N\\ n \end{array}\right)}$$

Then, the false discovery rate (FDR) correction was performed based on the *p*-value, and the function with a Q-value of ≤0.05 was generally considered to be significantly enriched. Here, *N* is the number of all genes annotated by GO, *n* is the number of DEGs in *N*, *M* is the number of all genes annotated with specific GO terms, and *m* is the number of DEGs in *M*.

### 2.6. Differentially Expressed Gene in the Susceptible and Resistant Strains of P. xylostella

The TPM method can eliminate the influence of different gene lengths and sequencing levels on gene expression calculation, and it can make the total expression level in different samples consistent. The TPM was calculated to represent the transcript-level expression [65]. The DESeq2 (v1.4.5) method is based on the negative binomial distribution principle, and differential expression analysis was performed using DESeq2 (v1.4.5) with Q value (Adjusted *p* value) ≤ 0.05, while the other parameters were the default values [66].

### 2.7. Real-Time Quantitative PCR Analysis of Gene Expression

qPCR was used to verify transcriptome sequencing results. Firstly, total RNA was extracted as described above from the fourth-instar larvae midguts. The genomic DNA was then removed using DNase I, and the concentration of total RNA was measured by NanoDrop 2000. The first-strand cDNA was synthesized from 2 ug RNA using “FastKing gDNA Dispelling RT SuperMix” (TianGen, Beijing, China) kit, following the manufacturer’s instructions. In addition, primer pairs (Additional file 3) were designed using Primer 5. PCR was conducted using “Eastep qPCR Master Mix” (Promega, Shanghai, China) on a CFX96 (BioRad, Hercules, CA, USA) sequence detection system. The qPCR mixture included 10 μL qPCR master Mix, 0.4 μL of each primer (10 μM), 7.2 μL of RNase-free Water, and 2 μL cDNA. The qPCR program started at 95 °C for 2 min, followed by a total of 40 cycles of 95 °C for 15 s and 60 °C for 50 s. In the melting curve analysis, an automatic dissociation step cycle was added to determine the specificity of primers. Relative quantification of genes was performed using the 2^−^^ΔΔCt^ method [67], and the expression level was normalized to the ribosomal L32 gene (GenBank: AB180441) [68]. For each treatment, we used three biological replicates and three technical replicates. Moreover, one-way ANOVA with Holm–Sidak’s test (*p* ≤ 0.05) was used to determine whether differences between treatments were statistically significant.

## 3. Results

### 3.1. Illumina Sequencing Analysis

Illumina high-throughput sequencing was performed on cDNA samples from midgut tissues of G88 and Cry1S1000 strains of *P. xylostella*. We tested a total of 12 samples using BGISEQ-500, and every sample produced an average of 6.35 Gb data. Upon mapping these reads to the *P. xylostella* DBM V2, we obtained a mapping rate from 51.94% to 53.66% of the compared genome and 61.56% to 65.02% of the compared genes. We defined low-quality reads as those with a mass value of less than 15 bases that accounted for more than 20% of the total number of bases in the reads (Table 1).

Moreover, the predicted new genes were 3641, and a total of 18,042 expressed genes were detected including 14,474 known genes and 3568 predicted new genes. In addition, 19,415 new transcripts were detected, of which 10,525 belonged to novel isoforms, 3641 were new protein-coding gene transcripts, and the remaining 5249 belonged to long noncoding RNAs (Table 2 and Table S1).

### 3.2. Gene Ontology (GO) Classification

**Figure 1:**
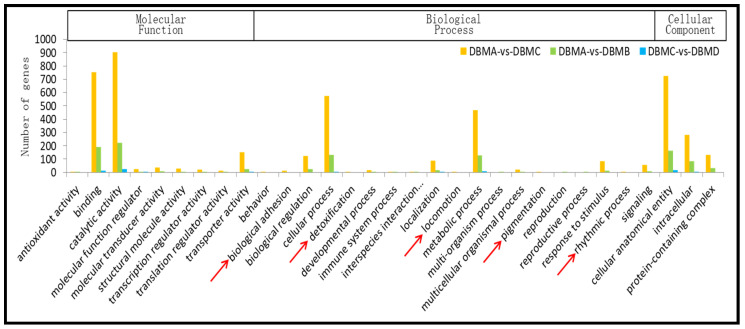
Histogram presentation of gene ontology (GO) classification between any two groups from susceptible, resistant, and two toxin-treated strains. The GO categories shown on the *X*-axis are divided into three main ontologies: molecular function, biological process, and cellular component. The *Y*-axis indicates the number of DEGs in each category. DBMA represents G88-susceptible strain; DBMB represents G88-susceptible strain with toxin treatment; DBMC represents Cry1S1000-resistant strain; DBMD represents Cry1S1000-resistant strain with toxin treatment. GO analysis showed that the distribution patterns of DEG function categories in each group were basically consistent, except for “biological adhesion”, “detoxification”, “locomotion”, “pigmentation”, and “rhythmic process” (red arrows), between susceptible and resistant strains.

Gene ontology (GO) classification was used to predict possible functions for the identified DEGs. The DEGs were assigned into 31 functional groups consisting of three categories (molecular function, biological process, and cellular component) based on the proposed GO function (Figure 1). Most were categorized as “binding”, “catalytic activity”, “transporter activity”, “biological regulation”, “cellular process”, “metabolic process”, “cellular anatomical entity”, “intracellular”, and “protein-containing complex”. In addition, a high proportion of DEGs were classified as “localization”, “response to stimulus”, and “signaling” (Figure 1). Among them, the DEGs were only assigned to “biological adhesion”, “detoxification”, “locomotion”, “pigmentation”, and “rhythmic process” between susceptible and resistant strains (DBMA-vs-DBMC).

### 3.3. Functional Classification by KEGG

**Figure 2:**
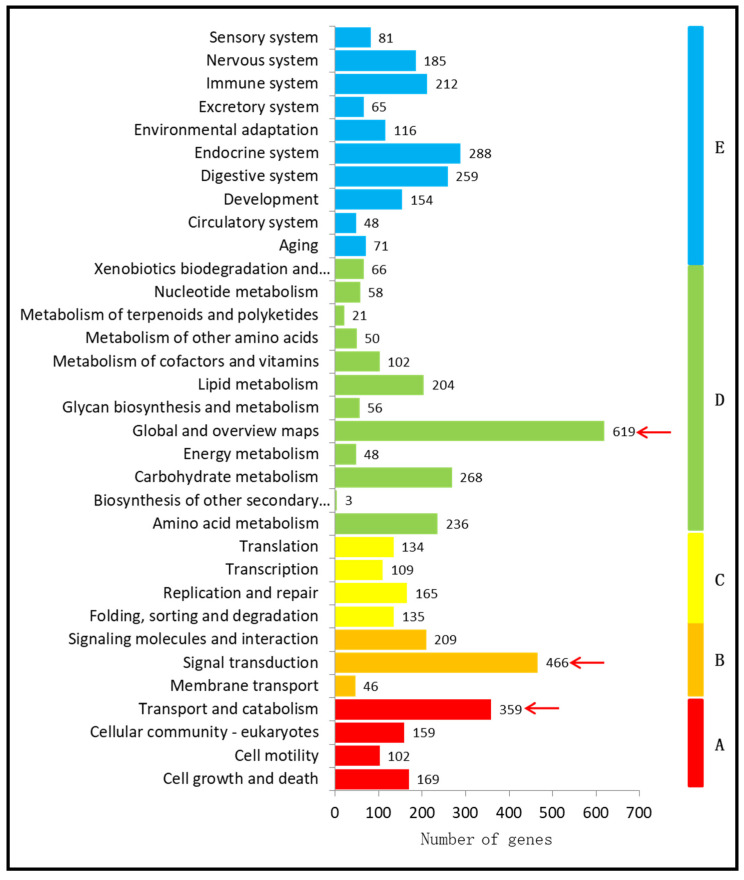
Histogram presentation of KEGG functional classification between G88 and Cry1S1000. The *X*-axis indicates the number of a specific category of genes, and the *Y*-axis is the enrichment of KEGG terms. Genes were classified into five branches: A—Cellular Processes; B—Environmental Information Processing; C—Genetic Information Processing; D—Metabolism; E—Organismal Systems. The red arrow indicates the pathway with more than 300 DEGs.

A KEGG pathway analysis was performed to obtain further insights into the global network. A total of 33 pathways were obtained between susceptible (G88) and resistant (Cry1S1000) strains, and they were distributed to five major groups: cellular components (789 DEGs), genetic information processing (543 DEGs), environmental information processing (721 DEGs), metabolism (1731 DEGs), and organismal systems (1479 DEGs). Among them, the majority of these DEGs were assigned to “global and overview maps” (619 DEGs), “signal transduction” (466 DEGs), and “transport and catabolism” (359 DEGs) (Figure 2). These KEGG annotations provide a meaningful resource for studying the functions, specific processes, and pathways in the resistance research of *P. xylostella*.

### 3.4. Differentially Expressed Genes (DEGs) in Midgut Transcripts

Before the analysis of DEGs among four groups, the PCA analysis was performed on the midgut samples collected by transcriptome sequencing using princomp in R software (v.3.6.1), which showed that the three samples from each group belonged to a separate cluster. This result suggests that the data were sufficient and reproducible (Figure S1).

**Figure 3:**
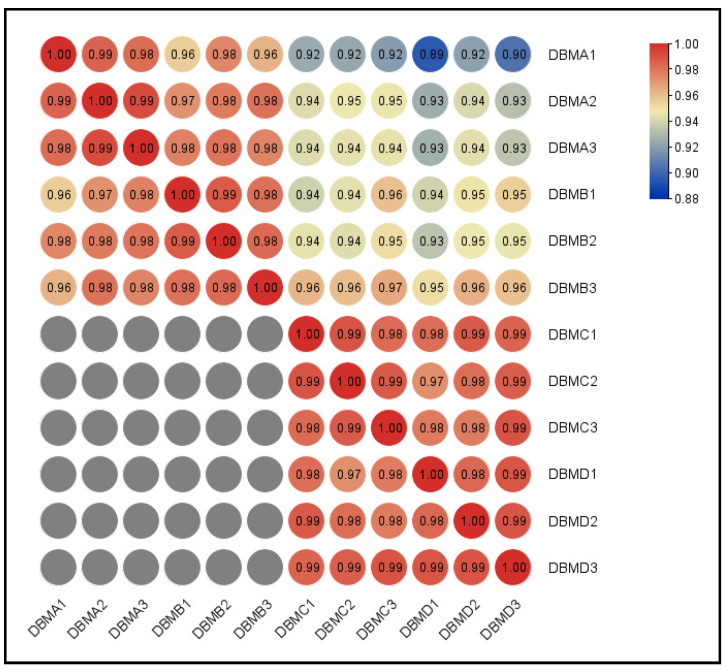
Pearson correlation coefficient heat map of gene expression between different samples. DBMA1, DBMA2, DBMA3: three biological repetitions from the susceptible strain (G88); DBMB1, DBMB2, DBMB3: three biological repetitions from the susceptible strain with toxin treatment (G88 + toxin); DBMC1, DBMC2, DBMC3: three biological repetitions from the resistant strain (Cry1S1000); DBMD1, DBMD2, DBMD3: three biological repetitions from the resistant strain with toxin treatment (Cry1S1000 + toxin).

Moreover, Pearson correlation analysis among samples showed that the *R^2^* values of biological replicates in each group were all greater than 0.98, which was much higher than 0.92 under ideal sampling and experimental conditions. Results showed that the similarity between the biological duplicates was very high, which met the ideal sampling requirements for the subsequent analysis of DEGs (Figure 3).

DEGs were then analyzed among the four treatment groups by comparing the midgut transcriptome. The results revealed 599, 3284, 4313, and 63 significant DEGs between DBMA-DBMB, DBMA-DBMC, DBMB-DBMD, and DBMC-DBMD in the midgut transcriptome of *P. xylostella* (Padj ≤ 0.05) (Figure S2).

**Figure 4:**
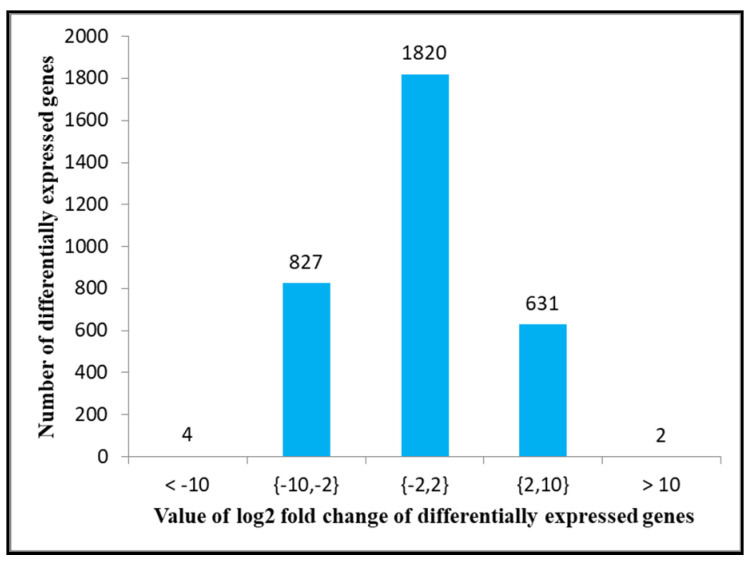
The number of differentially expressed genes with different values of log_2_ fold-change between susceptible (G88, DBMA) and resistant (Cry1S1000, DBMC) strains.

Among these, 1528 genes were up-regulated, and 1756 were down-regulated between G88 and Cry1S1000 strains. Among all the DEGs, 4 were down-regulated, 2 were up-regulated by more than 10 times, 827 were down-regulated, and 631 were up-regulated by 2 to 10 times (Figure 4).

### 3.5. Differentially Expressed Genes (DEGs) Involved in Insecticide Resistance

DEGs encoding Cry toxin receptors or participating in the Cry toxin pathway were further studied, including cadherin, aminopeptidase-N/P, alkaline phosphatase, ATP-binding cassette transporter family, heat shock protein, and V-type proton ATPase catalytic subunit A. Among them, only 4 DEGs for cadherin, 10 for aminopeptidase, 3 for alkaline phosphatase, 24 for ATP binding cassette transporter, and 7 for heat shock protein between G88 and Cry1S1000 strains were detected, all of which may be related to the resistance in *P. xylostella* (Table 3 and Table S2).

In this study, we examined not only DEGs that encode potential Cry toxin receptors but also DEGs that encode detoxification enzymes, such as carboxylesterase (CarEs), cytochrome P450 (P450s), glutathione S-transferase (GSTs), and some other genes related to metabolic insecticide resistance and insecticide targets. There were 24 DEGs encoding P450s, 15 of which were up-regulated, and 9 were down-regulated. In addition, 3 DEGs encoding CarEs were up-regulated, and the other 11 DEGs were down-regulated. The three DEGs encoding GST were all down-regulated (Table 3). Immune genes associated with hemolymph melanization, such as serpin protease and serpin protease inhibitor, were also detected. However, no serpin protease was found in the present study. Meanwhile, serpin protease inhibitor-related genes were searched, in which two of them were up-regulated and the other two were down-regulated compared with the G88-susceptible strain (Table 3).

### 3.6. Validation of Differentially Expressed Genes by qRT-PCR

**Figure 5:**
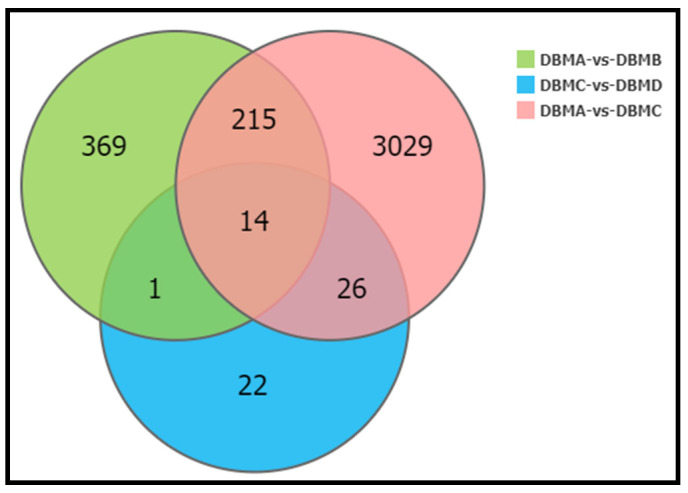
Venn diagram of differentially expressed genes between susceptible and resistant strains and two toxin-treated strains 24 h before sampling. DBMA represents G88-susceptible strain; DBMB represents G88-susceptible strain with toxin treatment; DBMC represents Cry1S1000-resistant strain; DBMD represents Cry1S1000-resistant strain with toxin treatment.

To eliminate the expression difference caused by toxin induction, we subtracted the DEGs between G88 and Cry1S1000 strains and the DEGs between these two strains and their toxin induction treatment group successively (DBMB, DBMD). Finally, we obtained a total of 3029 differentially expressed genes (Figure 5).

**Figure 6:**
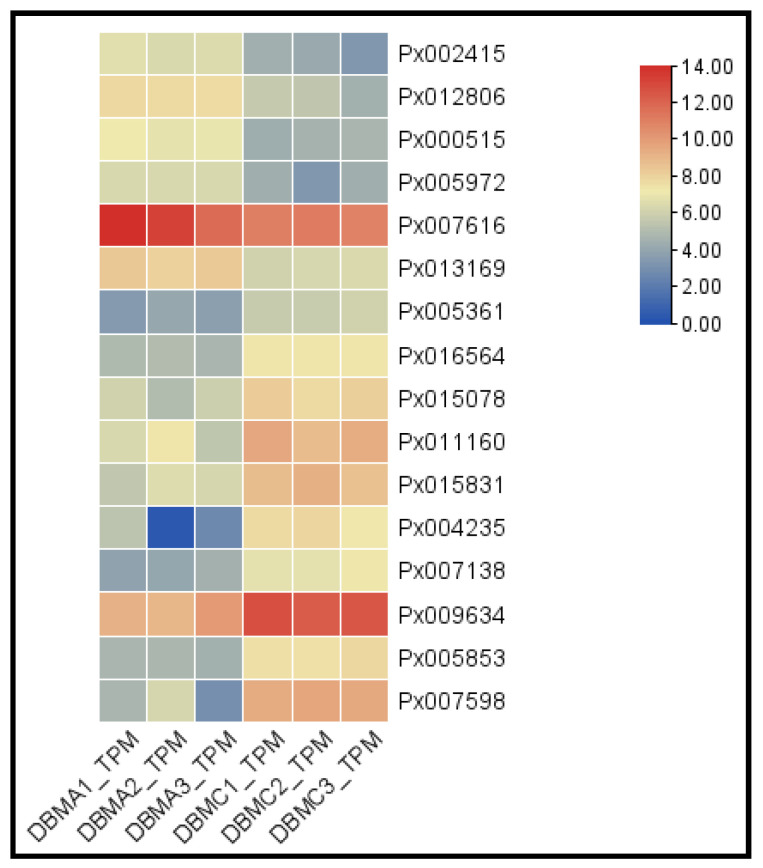
Heat maps of 16 genes with significant expression differences between the G88 and Cry1S1000 strains. DBMA_TPM and DBMC_TPM represent the value of G88 susceptible and Cry1S1000 resistant strains.

Firstly, we excluded the new genes predicted by BGI and the DEGs for which the TPM value was less than 10. Thus, after the exclusion step, 376 DEGs were left. Then, we carried out subsequent analysis combined with the log_2_ fold-change, in which we retained the DEGs having an absolute value of greater than 2 log_2_ fold-change. Thus, we obtained a total of 16 differential candidate genes, of which 6 genes were down-regulated, and the log_2_ fold-change was less than or equal to −2, while 10 genes were up-regulated and the log_2_ fold-change was greater than or equal to 2 (Table 4, Figure 6).

**Figure 7:**
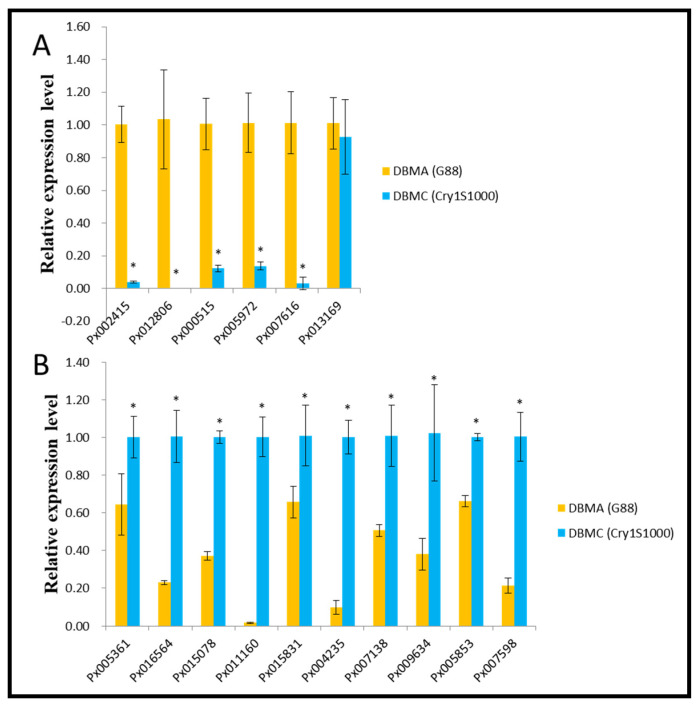
qRT-PCR analysis of differentially expressed genes to confirm the expression patterns indicated by sequencing. (**A**): 6 down-regulated DEGs, (**B**): 10 up-regulated DEGs. Data are shown as mean ± standard deviation (SD) and asterisk indicates significant difference (*p* < 0.05).

To verify the expression patterns of genes, we selected 16 DEGs (including 6 down-regulated DEGs and 10 up-regulated DEGs) for qRT-PCR. In addition, the ribosomal L32 gene (GenBank: AB180441) was set as the candidate reference gene for qRT-PCR normalization. The results showed that the transcriptome expression pattern was consistent with the qRT-PCR results (Figure 7).

## 4. Discussion

*P. xylostella* is a highly destructive vegetable pest, and its resistance has badly been affecting the continuable use of insecticides. However, the resistance mechanism of *P. xylostella* to Cry1Ac remains to be improved. In this study, transcriptome analysis was performed on Cry1Ac-susceptible (DBMA) and -resistant (DBMC) strains and two toxin-treated (DBMB, DBMD) strains 24 h before sampling. A total of 12 samples were examined using the BGISEQ-500 platform, and every sample produced an average of 6.35 Gb of clean data. In addition, 3284 DEGs were detected between susceptible (DBMA) and resistant (DBMC) strains, 599 DEGs between susceptible (DBMA) and its toxin-treated strain (DBMB), and 63 DEGs between the resistant strain (DBMC) and its toxin-treated strain (DBMD). Among them, DEGs encoding Bt Cry toxin receptors cadherin, GPI-anchored alkaline phosphatase (ALP), glycosylphosphatidylinositol (GPI)-anchored aminopeptidase N (APN), and ATP-binding cassette transports (ABC) were investigated in detail. Furthermore, DEGs associated with metabolic insecticide resistance and insecticide targets and immune genes related to hemolymph melanization were also detected. Presumably, all the significantly differentially expressed genes detected above may be associated with the resistance to Bt toxin Cry1Ac.

Among all the DEGs, five were annotated as cadherin: four of them were up-regulated in the resistant strain, and only one was down-regulated. In addition, no DEGs were up-regulated in the toxin-treated susceptible (DBMB) strain compared with the G88-susceptible strain (DBMA), which is consistent with the previous finding that the cadherin-like gene is not associated with resistance to Bt toxin Cry1Ac in *P. xylostella* [69]. Baxter et al. [70] concluded that the resistance of *P. xylostella* to Cry1Ac was not related to the mutation of cadherin but through other mechanisms. This finding contradicts the correlation between cadherin mutation and Cry1Ac resistance in *Heliothis virescens* and *Pectinophora gossypiella*. After analyzing the genetic mapping of resistance genes already reported in other species on *P. xylostella*, they also [71] found that these genes were not on the chromosome where the Cry1Ac resistance locus was located. In addition, the bioassay results of the knockout G88 susceptible strain of *P. xylostella* after the mutation of cadherin showed no significant difference in resistance ratio (unpublished). Unlike other lepidopteran insects, *P. xylostella* itself is complex and changeable, with many SNPs, which increases the difficulty of studying the resistance mechanism of *P. xylostella* to Bt by analogy. Therefore, it remains to be further investigated whether the orthologous genes of cadherin will play a role in resistance.

Aminopeptidase is a peptide chain end-hydrolase and is usually divided into four categories: N (AP-N; EC3.4.11.7), A (AP-A; EC3.4.11.2), P (AP-P; EC3.4.11.9), and W (AP-W; EC3.4.11.16) [72]. Aminopeptidase can catalyze the splitting of the amino-terminal residues of many proteins, and it is widely present in animals and plants. Among them, APN is the first reported receptor protein of Bt toxin. Since then, the APN of many lepidopteran insects has been identified as a binding protein of Bt toxin, which plays an essential role in insect resistance. Knight et al. [73] cloned the Cry1Ac toxin receptor protein gene in the midgut of the *Manduca sexta* and found that the protein was encoded by APN. Similarly, Gill et al. [74] used the same method and successfully found the receptor protein APN for the Bt toxin on the *Heliothis virescen*. In addition, the toxin binding test found that APN is the Cry1Ac toxin-binding receptor for *P. xylostella* [75], *Lymantria dispa* [76,77], *Bombyx mori* [78], and *Trichoplusia ni* [79]. Tiewsiri and Wang [14] found that Bt Cry1Ac resistance was related to the down-regulation of *APN1* but not with the up-regulation of *APN6* in cabbage loopers. Qiu et al. [80] demonstrated that RNA interference knockdown of APN genes decreases the susceptibility of *Chilo suppressalis* larvae to Cry1Ab/Cry1Ac and Cry1Ca-expressing transgenic rice. A recent study also found that knockdown of the APN genes decreases the susceptibility of *C. suppressalis* larvae to Cry1Ab/Cry1Ac and Cry1Ca [16]. In this study, 14 DEGs were annotated as aminopeptidases between susceptible and resistant strains, among which only two were up-regulated. Twelve DEGs encoding aminopeptidases were down-regulated compared with the G88 strain.

Another GPI-anchored Cry receptor involved in Bt resistance is alkaline phosphatase (ALP). In this study, we did not find any differentially expressed genes annotated as ALP. Another important group of receptors involved in Cry1Ac resistance is the ATP-binding cassette transporters. Many previous studies have proven that resistance to Cry1Ac is related to ABC transports. Among them, the structural variation or expression changes of *ABCA*, *ABCB*, *ABCC*, *ABCG*, and *ABCH* are all related to Bt resistance [29,31,33,34,40,81,82,83,84,85,86]. The results from Yang [34] confirmed that ABCA2 is essential for the toxicity of Cry2Ab in *T. ni*, whereby mutation of *ABCA2* confers resistance to Cry2Ab in the resistant *T. ni* strain derived from a Bt-resistant greenhouse population. In addition, Niu et al. [81] demonstrated that heterologous expression of *DvABCB1* in Sf9 and HEK293 cells conferred sensitivity to the cytotoxic effects of Cry3A toxins in Western corn rootworm (WCR), *Diabrotica virgifera verifier.* Tian et al. [82] found that elevated expression of *PxPgp1* was observed in *P. xylostella* after they were exposed to abamectin treatment. Zhou et al. [83] also found that reduced expression of the *P-glycoprotein* gene *PxABCB1* is linked to resistance to Bt Cry1Ac toxin in *P. xylostella* (L.). Tanaka et al. [29] demonstrated Cry toxin receptor functionality for *ABCC2* and highlighted the crucial role of this protein and cadherin. Meanwhile, bioassays of CRISPR-based mutant strains demonstrated that the deletion of *PxABCC2* or *PxABCC3* alone provided <4-fold tolerance to Cry1Ac, while disruption of both genes together conferred >8000-fold resistance to Cry1Ac, suggesting the redundant or complementary roles of *PxABCC2* and *PxABCC3* in *P. xylostella* [33]. Furthermore, RNA interference (RNAi)-mediated suppression of *Pxwhite* gene expression significantly reduced larval susceptibility to Cry1Ac toxin. Genetic linkage analysis confirmed that down-regulation of the *Pxwhite* gene is tightly linked to Cry1Ac resistance in *P. xylostella*. Additionally, silencing *PxABCH1* by a relatively high dose of dsRNA dramatically reduced its expression and resulted in larval and pupal lethal phenotypes in both susceptible and Cry1Ac-resistant *P. xylostella* strains [31]. In addition, Zhou et al. [83] found that *P-glycoprotein* gene *PxABCB1* was significantly down-regulated in two resistant strains through preliminary transcriptome analysis. More importantly, knockdown of this gene in susceptible strain DBM1AC-S led to a significant reduction in sensitivity. Thus, they concluded that the decrease in *PxABCB1* expression was closely related to Cry1Ac resistance. Xu et al. [87] constructed a stable *PxABCC2* cell line and found that cell lines with stable *PxABCC2* were significantly less sensitive to avermectin and chlorfenapyr compared to the control group. Their study showed that the upregulation of *PxABCC2* gene was associated with insecticide resistance. This study also provides new insights into the cross-resistance between Bt toxins and chemical insecticides.

We also counted the number of different genes related to insecticide resistance in each group, as shown in Table 3. In the follow-up study, we conducted a functional verification of whether the CYP gene is involved in Bt resistance mechanism. The results provide new information on the interaction between Bt resistance and chemical insecticides. This study found 14 differentially expressed genes belonging to the ABC family between susceptible and resistant strains. Among them, five were up-regulated, and nine were down-regulated. Whether these genes are also involved in Bt resistance remains to be further studied. Our study provides rich candidate gene resources for the study of transporter involvement in Bt resistance.

The change of midgut protease-induced protoxin activation is one of the proposed mechanisms associated with Bt resistance, with some controversial evidence reported previously. For example, Gong et al. [88] found dramatically decreased activities of casein lysase and trypsin in the resistant *P. xylostella* strain SZ-R, due to significantly down-regulated expression of *PxTryp_SPc1*. A comparable finding, significantly lowered expression of *PxTryp_CFT1* (*Px007616*) in the resistant *P. xylostella* strain CryS1000, was also identified in this study. However, Wei et al. [89] demonstrated that the LF120 strain (resistant strain) of *H. armigera* developed strong resistance to both activated toxin and protoxin, and the use of protease inhibitors did not change the LC_50_ of the resistant strain to Cry1Ac protoxin. The role of midgut protease-induced protoxin activation, therefore, is well worth further investigation.

Our results also revealed DEGs encoding heat-shock proteins. A previous study showed that PxHsp90 assists Cry1A proteins by enhancing their binding to the receptor and protecting Cry protoxins from gut protease degradation [39]. In this study, five DEGs encoding heat-shock proteins were found between the susceptible and resistant strains. Among them, one was up-regulated, and four were down-regulated. Meanwhile, genes encoding serpin proteases and serpin protease inhibitors were involved in the immune function to regulate hemolymph melanization. However, we only found one DEG between G88 and Cry1S1000 strains.

Genes encoding the detoxification enzymes of carboxylesterase (CarE), cytochrome P450 (P450), and glutathione S-transferase (GST) were also identified. Among them, P450 family members play a significant role in the degradation of chemical insecticides. In this study, 24 DEGs were identified as P450s, 4 were CarEs, and 4 were GSTs. A previous study showed that RNA interference-mediated knockdown of *CYP6BG1* from *P. xylostella* reduced larval resistance to permethrin [90]. In addition, Chen [91] demonstrated that the overexpression of three CYP genes, *CYP6CY14*, *CYP6CY22*, and *CYP6UN1,* contributed to dinotefuran resistance in *Aphis gossypii*. Furthermore, knockdown of *CYP4PR1* increased the susceptibility to deltamethrin in pyrethroid-resistant insects [92]. A dual-Luciferase Reporter assay, a yeast one-hybrid (Y1H) assay, and RNA interference confirmed that the mutation of the *PxABCG1* promoter in the resistant strain resulted in Bt being unable to combine with the toxin, resulting in strong resistance [93]. Qin et al. [94] showed that MAPK-activated *PxJun* inhibited the expression of *PxABCB1*, leading to the resistance of *P. xylostella* to Cry1Ac. Their study is the first to identify transcription factors involved in the transcriptional regulation of Cry receptor genes in the gut of Bt-resistant insects. Similar results were also found in several CYP genes found in our transcriptome data. Furthermore, preliminary sequence verification found that there were significant variations in the promoter regions of several CYP genes between susceptible and resistant strains. Notably, it is widely reported that the P450 gene is mainly related to insecticide resistance. However, whether or not the P450 gene is involved in the detoxification metabolism of Bt toxin, and what role it plays in this process, needs further validation.

To find other resistance-related genes, we analyzed the GO function and KEGG function classifications for DEGs. The analysis of the GO function classification showed that 1932 genes were assigned to molecular functions, among which “binding”, “catalytic activity”, and “transporter activity” had the largest number. Furthermore, 1461 genes were assigned to biological processes and 1138 genes to cellular components. Specifically, “cellular process” and “metabolic process” categories in the biological process domain were represented by 574 and 468 DEGs between G88 and Cry1S1000 strains, respectively. In the cellular component domain, the top three categories were “cellular anatomical entity”, “intracellular”, and “protein-containing complex”, and the combined total number of DEGs was more than 1100. Therefore, future work should be focused on DEGs under these categories, specifically searching genes related to Bt resistance. In our study, a large number of genes were significantly enriched in the “hydrolase activity”, “catalytic activity”, “metabolic process”, “primary metabolic process”, and “organic substance metabolic process”-related GO categories (Figure S3).

Meanwhile, the KEGG pathway analysis indicated that 466 DEGs were annotated as “signal transduction” under the environmental information processing. In the metabolism domain, 619 DEGs were annotated as “global and overview maps”, representing the largest number of DEGs between G88 and Cry1S1000 strains in the KEGG function classification. This result suggests that there might be many genes related to Bt resistance in this category. In addition, DEGs annotated as “transport and catabolism”, “digestives system”, and “endocrine system” also need further investigation in future research. KEGG category analysis revealed a significant range of enrichment genes in “fanconi anemia pathway”-related KEGG pathways (Figure S4).

After continuous laboratory screening with activated Cry1Ac toxin for 20 generations, the SZ strain that originally had only 20 times resistance to Cry1Ac reached a 1200 times resistance level. Moreover, studies showed that resistance of the SZBT strain to Cry1Ac was controlled by a single, autosomal, and incomplete recessive gene. This study provides good material for studying the mechanism of Bt resistance at single loci [95]. At the same time, it also shows that the change of resistance may be relatively simple under laboratory selection methods. In particular, we can find Bt resistance genes with stronger specificity through this method to analyze the mechanism of Bt resistance. The G88 susceptible strain used in this paper has been raised for more than 110 generations in the laboratory, and the Cry1S1000 strain has been raised for more than 90 generations. Stable indoor breeding strains provide good experimental materials for subsequent studies, and they also provide more possibilities for the search of Bt resistance genes.

In the previous study [43], a comparative transcriptomic analysis was conducted between a susceptible strain (MM) and two resistant strains (GK and MK) of *P. xylostella*, of which the sources of MM and MK were consistent with ours. However, Lei et al. continuously exposed resistant strains to Bt toxin to maintain their resistance multiples. The resistant strain (Cry1S1000) used in this study [33] was screened with Cry1Ac toxin only once when they first arrived in our laboratory, and then it was bred and passed on in an environment without toxin exposure until now. Due to the selective induction of Bt toxin resistance genes, we can actually understand these two resistance genes as different resistant lines; that is, when we compare them with a susceptible strain at the same time, the DEGs should be different. In addition, when we verified the function of the same gene (*PxABCC2* and *PxABCC3*), we obtained completely different results [33,56], so it is reasonable for us to compare two completely different resistance strains. As shown in Table 3, we obtained a large number of CYP genes. However, in subsequent functional verification experiments (unpublished), we found sequence differences in coding regions and promoter regions of multiple CYP genes between susceptible and resistant strains, which may be related to resistance differences between the two strains. Unlike the previous studies [43], before sampling we performed a short 24 h induction process for transcriptome sequencing using low concentrations of Bt toxin in both resistant and susceptible strains. This process will eliminate some differences in gene transcription levels due to toxin induction, thereby gaining more constitutive types of DEGs between both strains.

## 5. Conclusions

In conclusion, this study reveals many DEGs between G88-susceptible and Cry1S1000-resistant strains. In view of our results, several factors may participate in *P. xylostella* resistance to Cry1Ac toxins. The widely reported cadherin and aminopeptidase N, alkaline phosphatase, and ATP-binding cassette transporter may be involved in Cry1Ac resistance. Among them, we have shown 14 DEGs encoding ABC transporters between G88 and the Cry1S1000 strains, suggesting that genes from the ABC family are more likely to play an important role in Bt resistance. Moreover, DEGs related to detoxification metabolism may account for *P. xylostella* resistance to the Cry1Ac toxins, such as P450s (24 DEGs), CarEs (4 DEGs), and GSTs (4 DEGs). These important genetic resources, coupled with ongoing antibody analysis resources, are more conducive to screening and obtaining genes related to Cry1Ac resistance in *P. xylostella*. RNAi or CRISPR/Cas9 technologies could be used to verify relationships between these candidate genes and Bt resistance.

## Figures and Tables

**Table 1:** Summary of the midgut transcriptome reads of *P. xylostella.* DBMA1, DBMA2, DBMA3: three sample replications from the susceptible strain (G88); DBMB1, DBMB2, DBMB3: three sample replications from the susceptible strain with toxin treatment (G88 + toxin); DBMC1, DBMC2, DBMC3: three sample replications from the resistant strain (Cry1S1000); DBMD1, DBMD2, DBMD3: three sample replications from the resistant strain with toxin treatment (Cry1S1000 + toxin).

Samples	Raw Reads (M)	Clean Reads (M)	Q20 (%) ^a^	Q30 (%) ^b^	Clean Reads Ratio (%)
G88_1 (DBMA1)	65.18	61.75	97.49	90.02	94.74
G88_2 (DBMA2)	67.68	62.43	97.00	88.62	92.24
G88_3 (DBMA3)	67.68	61.70	96.58	87.38	91.16
G88 + toxin_1 (DBMB1)	65.18	60.99	96.77	87.91	93.58
G88 + toxin_2 (DBMB2)	67.68	64.15	97.30	89.57	94.78
G88 + toxin_3 (DBMB3)	67.68	63.14	96.70	87.78	93.29
Cry1S1000_1 (DBMC1)	70.19	66.30	97.00	88.61	94.47
Cry1S1000_2 (DBMC2)	67.68	63.07	96.68	87.87	93.19
Cry1S1000_3 (DBMC3)	67.68	64.06	97.38	89.74	94.65
Cry1S1000 + toxin_1 (DBMD1)	67.68	62.53	96.60	87.61	92.39
Cry1S1000 + toxin_2 (DBMD2)	70.12	66.35	97.36	89.71	94.62
Cry1S1000 + toxin_3 (DBMD3)	70.18	65.73	97.25	89.19	93.65

^a^ Ratio of the number of bases with mass value greater than 20 to the total number of reads after filtration. ^b^ Ratio of the number of bases with mass value greater than 30 to the total number of reads after filtration.

**Table 2:** Statistical summary of new transcript types.

Total Novel Transcripts	Coding Transcripts	Novel Transcripts	Novel Isoforms	Novel Genes
19,415	14,166	5249	10,525	3641

**Table 3:** The number of differentially expressed genes potentially participated in Cry1Ac resistance in *P. xylostella*. DBMA, DBMB, DBMC, and DBMD represent the strains of G88, G88 with toxin treatment, Cry1S1000, and Cry1S1000 with toxin treatment.

Genes	DBMA/DBMC	DBMA/DBMB	DBMC/DBMD
Bt resistance			
Cadherin	5	0	0
Aminopeptidase N/P	14	4	0
Alkaline phosphatase	0	0	0
ABC transporter	14	3	0
Trypsin	55	22	1
Glycolipid	0	0	0
Heat-shock proteins	5	6	0
Insecticide targets and metabolic insecticide resistance			
Cytochrome P450 (P450s)	24	7	3
Carboxylesterase (CarEs)	4	0	0
Glutathione S-transferase (GSTs)	4	3	1
Acetylcholinesterase	2	1	0
Nicotinic acetylcholine receptor	0	0	0
GABA receptor	0	0	0
Glutamate receptor	10	1	0
G-protein coupled receptor	3	0	0
Ryanodine receptor	1	0	0
Sodium channel	1	1	0
Chloride channel	1	0	0
Immune-related genes			
Serpin protease	0	0	0
Serpin protease inhibitor	1	0	0

**Table 4:** Summary of the most significant 16 differentially expressed genes between susceptible and resistant strains. DBMA-TPM represents the TPM value of G88-susceptible strain, and DBMC-TPM represents the TPM value of Cry1S1000-resistant strain.

	Gene ID	DBMA-TPM	DBMC-TPM	Log_2_ (DBMC/DBMA)	Annotation	Up/Down
1	Px002415	92.57	15.99	−2.53	Probable multidrug resistance-associated protein lethal (2) 03659	Down
2	Px012806	217.58	39.78	−2.45	Luciferin 4-monooxygenase	Down
3	Px000515	127.45	23.13	−2.46	Esterase FE4	Down
4	Px005972	82.80	16.80	−2.30	N-acetylneuraminate lyase	Down
5	Px007616	9973.06	2139.83	−2.22	Trypsin CFT-1	Down
6	Px013169	314.29	77.30	−2.02	Lactase-phlorizin hydrolase	Down
7	Px005361	12.89	58.54	2.18	Probable glutamine-dependent NAD(+) synthetase	Up
8	Px016564	29.39	165.13	2.49	Hydroxyacyl-coenzyme A dehydrogenase, mitochondrial	Up
9	Px015078	53.31	268.45	2.33	Ecdysteroid UDP-glucosyltransferase	Up
10	Px011160	96.75	630.73	2.70	Lactase-phlorizin hydrolase	Up
11	Px015831	71.64	488.08	2.77	Zinc carboxypeptidase A 1	Up
12	Px004235	15.75	204.14	3.70	Putative uncharacterized protein	Up
13	Px007138	17.01	125.17	2.88	Leucine-rich repeat- containing protein C10orf11 homolog	Up
14	Px009634	734.96	6072.207	3.04	Ecdysteroid-regulated protein	Up
15	Px005853	24.69	200.48	3.02	NADH dehydrogenase [ubiquinone] 1 beta subcomplex subunit 2, mitochondrial	Up
16	Px007598	36.14	750.73	4.38	Chymotrypsin−1	Up

## Data Availability

The datasets presented in this study can be found in online repositories. The names of the repository/repositories and accession number(s) can be found below: NCBI SRA; PRJNA768117.

## Supplementary Materials

The following are available online at https://www.mdpi.com/article/10.3390/insects12121091/s1, Figure S1. PCA analysis of midgut samples of *P. xylostella*; Figure S2. The number of differentially expressed genes between groups; Figure S3. GO enrichment of differentially expressed genes between G88 susceptible (DBMA) and Cry1S1000 resistant (DBMC) strains; Figure S4. KEGG enrichment of differentially expressed genes between G88 susceptible (DBMA) and Cry1S1000 resistant (DBMC) strains; Table S1. Gene ID and annotation information of the novel transcript; Table S2. Gene ID of differentially expressed genes between groups; Table S3. Primer sequences for qRT-PCR of 19 selected differentially expressed genes.
