# Supplementary material for: A Novel Reference for Bt-Resistance Mechanism in Plutella xylostella Based on Analysis of the Midgut Transcriptomes
Source: Insects. 2021 Dec 7;12(12):1091. doi: 10.3390/insects12121091 (PMC8708430; doi:10.3390/insects12121091)

Figure S1. PCA analysis of midgut samples of *P. xylostella*. DBMA1, DBMA2, DBMA3: three sample replications from the susceptible strain (G88); DBMB1, DBMB2, DBMB3: three sample replications from the susceptible strain with a toxin treated (G88+toxin); DBMC1, DBMC2, DBMC3: three sample replications from the resistant strain (Cry1S1000); DBMD1, DBMD2, DBMD3: three sample replications from the resistant strain with a toxin treated (Cry1S1000+toxin).

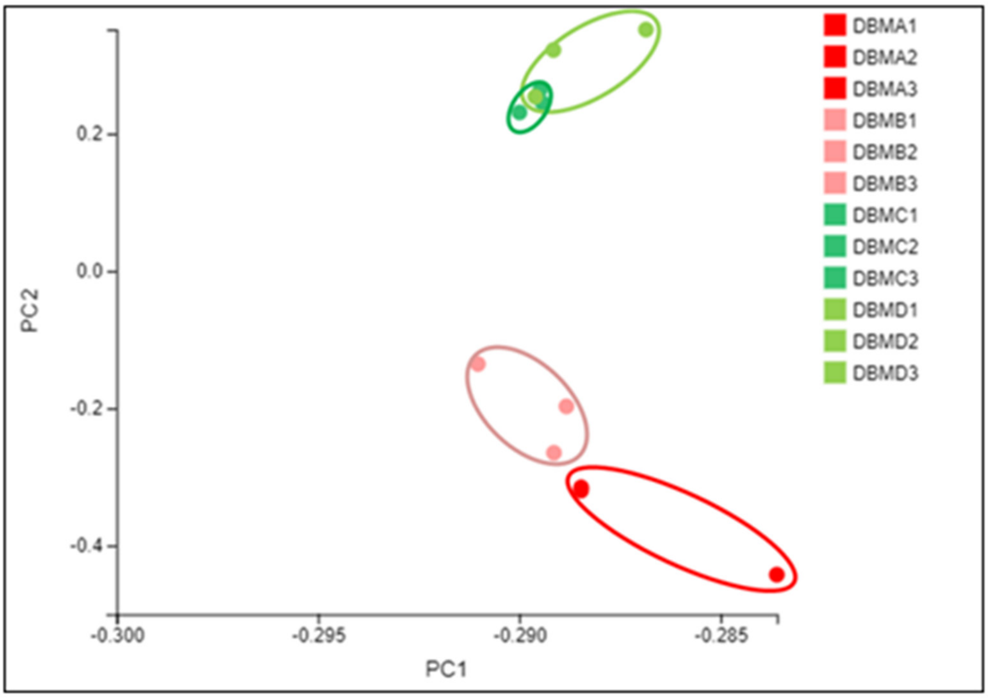

Supplement: Supplementary file 1 [file insects-12-01091-s001.zip › Figure S1.pdf]
