# Supplementary material for: A Novel Reference for Bt-Resistance Mechanism in Plutella xylostella Based on Analysis of the Midgut Transcriptomes
Source: Insects. 2021 Dec 7;12(12):1091. doi: 10.3390/insects12121091 (PMC8708430; doi:10.3390/insects12121091)

Figure S2. The number of differentially expressed genes between groups. DBMA represents G88 sus-ceptible strain; DBMB represents G88 susceptible strain with toxin treated; DBMC represents Cry1S1000 resistant strain; DBMD represents Cry1S1000 resistant strain with toxin treated.

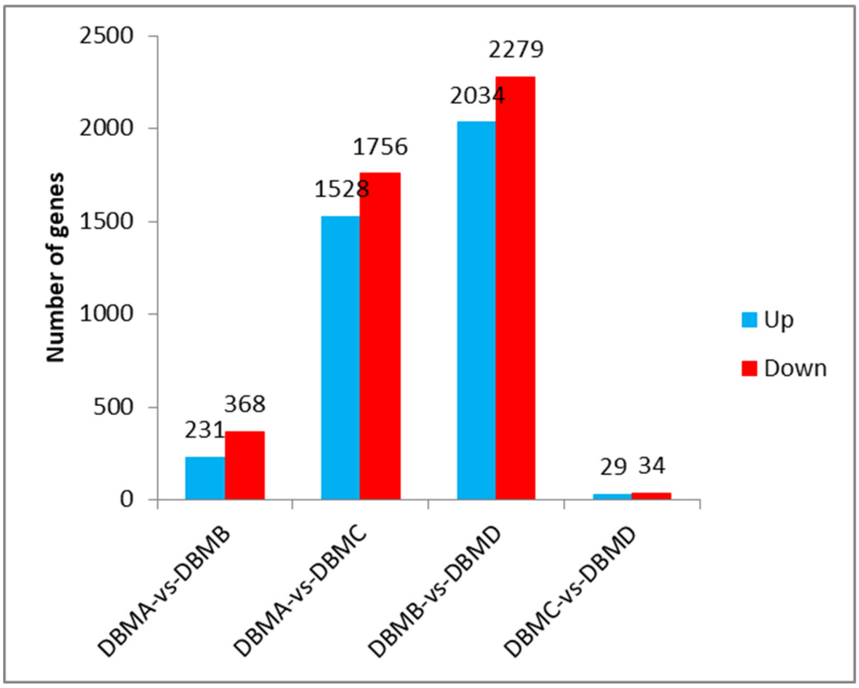

Supplement: Supplementary file 1 [file insects-12-01091-s001.zip › Figure S2.pdf]
