# Supplementary material for: A Novel Reference for Bt-Resistance Mechanism in Plutella xylostella Based on Analysis of the Midgut Transcriptomes
Source: Insects. 2021 Dec 7;12(12):1091. doi: 10.3390/insects12121091 (PMC8708430; doi:10.3390/insects12121091)

Figure S4. KEGG enrichment of differentially expressed genes between G88 susceptible (DBMA) and Cry1S1000 resistant (DBMC) strains.

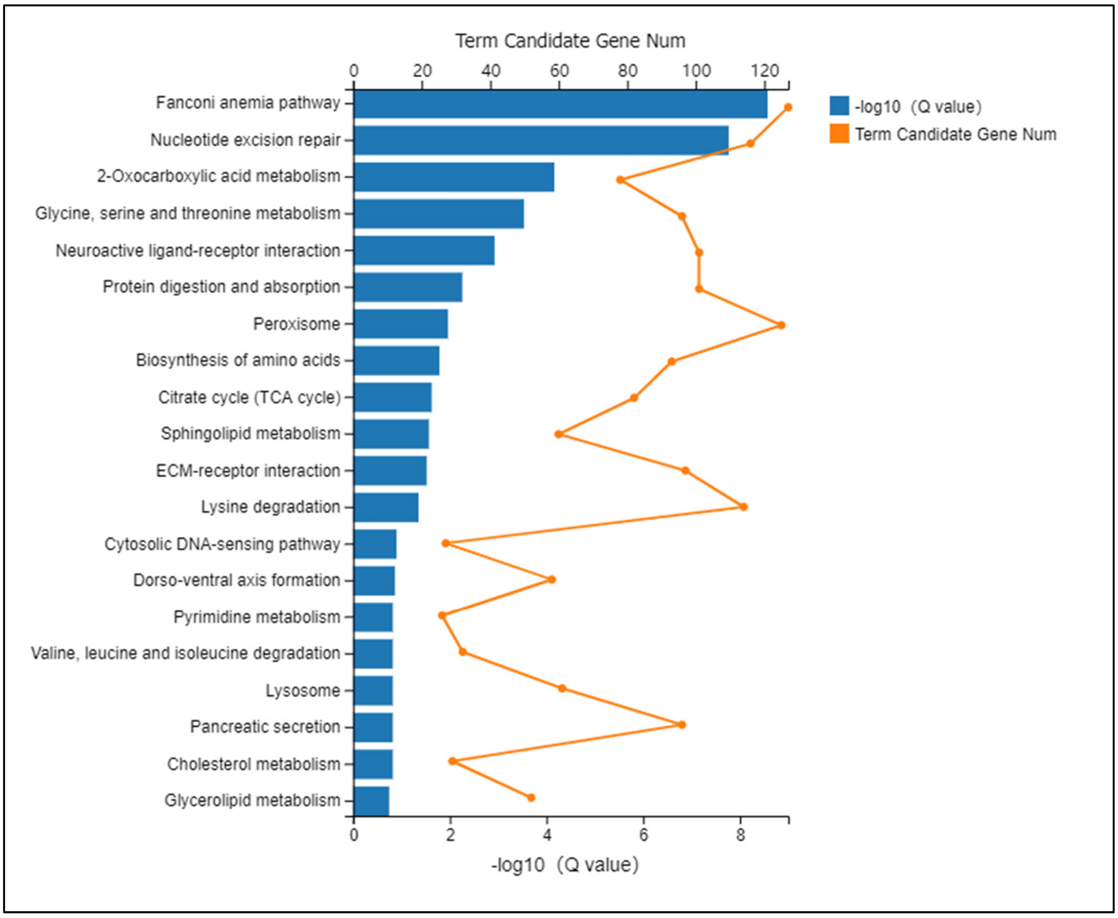

Supplement: Supplementary file 1 [file insects-12-01091-s001.zip › Figure S4.pdf]
