# Supplementary material for: A Novel Reference for Bt-Resistance Mechanism in Plutella xylostella Based on Analysis of the Midgut Transcriptomes
Source: Insects. 2021 Dec 7;12(12):1091. doi: 10.3390/insects12121091 (PMC8708430; doi:10.3390/insects12121091)
Supplement: Supplementary file 1 [file insects-12-01091-s001.zip › Table S3.pdf]

Table S3. Primer sequences for qRT-PCR of 26 selected differentially expressed genes.

Gene	Primer	Primer sequence (5'-3')
Px002415	qT1-F	TTCCTCACCATCGTCACCTACG
	qT1-R	GAACAGCTCGGCGGTGAAG
Px012806	qT2-F	CTTGGCGCTATGCTGAGTGG
	qT2-R	TTGATGCTCGTTTCGTGAGC
Px000515	qT3-F	ATTCCGTATGCTGCTCCACC
	qT3-R	ATCGTACTGGTGGCAATTAGGC
Px005972	qT4-F	GCGACTGGACTGCATATAATGGTG
	qT4-R	GGTGGGCTTGAAGTACAGCTC
Px007616	qT5-F	TGCCGGATGGGGACACAC
	qT5-R	CCCGAGCAGAGCATGTTGTC
Px013169	qT6-F	CTTCCTCTTCGGAGCTGGC
	qT6-R	GCTGCCGTCCTGGGCTATC
Px009394	qT7-F	GACCGAGGATAATGTACCTCTTATACAG
	qT7-R	CTTGGCGCACGATGGTTTCC
Px016486	qT8-F	TGCAGACATTCCTGGCGGC
	qT8-R	CAGCGCCCACCACTATGAAG
Px005361	qT9-F	TGGCAGTTCCGGGCCATC
	qT9-R	TGGGTTTGCTCACAACCTGC
Px016564	qT10-F	TTCGTGGTCAACCGCCTGC
	qT10-R	GTGTAGTCCGCTAGCTCGAAGG
Px015078	qT11-F	AACGTAAGATTAGAGAAGTGGCTGC
	qT11-R	CAATCAACGGTACATTCGCTGC
Px011160	qT12-F	ATGAGCCTGCAAATCCTAACTCTG
	qT12-R	TAGTTCCTTGGGGAATCCACCG
Px015831	qT13-F	GGGACACCTACTACGACTATGAAGC
	qT13-R	CGGGTTGTAGAGATCCTCACGTAC
Px004235	qT14-F	GTGGAACCCCAAGCGGAG
	qT14-R	CGTTGACAAGCACCGTCCAC
Px007138	qT15-F	CGGCAGCACCCAGTTACTTC
	qT15-R	TTCTGCCCGCTGGTCCTC
Px009634	qT16-F	CTCACGCAGCTGAAGGACG
	qT16-R	TCCTGGCCCAGCGGGTAG
Px005853	qT17-F	GACCGTTGCACCCATTTGC
	qT17-R	CTTCATCTGCACACATGCGC
Px000506	qT18-F	GAGGCGGTACTAGAAGCTCAAG
	qT18-R	CCGACGGATGCCCTTTATCAC
Px007598	qT19-F	AAGGACCGTGCTCACCGC
	qT19-R	GTCACGAACCTCTCGAACGC
RPL32	RPL32-F	CCAATTTACCGCCCTACC
	RPL32-R	TACCCTGTTGTCAATACCTCT
